# Phosphor-free white-light emitters using *in-situ* GaN nanostructures grown by metal organic chemical vapor deposition

**DOI:** 10.1038/srep17372

**Published:** 2015-12-02

**Authors:** Daehong Min, Donghwy Park, Jongjin Jang, Kyuseung Lee, Okhyun Nam

**Affiliations:** 1 Convergence Center for Advanced Nano Semiconductor (CANS), Department of Nano-Optical Engineering, Korea Polytechnic University (KPU), Siheung-si, Gyeonggi-do 429-793, Republic of Korea

## Abstract

Realization of phosphor-free white-light emitters is becoming an important milestone on the road to achieve high quality and reliability in high-power white-light-emitting diodes (LEDs). However, most of reported methods have not been applied to practical use because of their difficulties and complexity. In this study we demonstrated a novel and practical growth method for phosphor-free white-light emitters without any external processing, using only *in-situ* high-density GaN nanostructures that were formed by overgrowth on a silicon nitride (SiN_x_) interlayer deposited by metal organic chemical vapor deposition. The nano-sized facets produced variations in the InGaN thickness and the indium concentration when an InGaN/GaN double heterostructure was monolithically grown on them, leading to white-color light emission. It is important to note that the *in-situ* SiN_x_ interlayer not only facilitated the GaN nano-facet structure, but also blocked the propagation of dislocations.

Indium gallium nitride (InGaN) based white LEDs are widely used as lighting and back light units (BLUs) in displays. These LEDs are generally manufactured by adding yttrium aluminum garnet (YAG) yellow phosphor on top of high-power blue LED chips to enable white light emission. However, wavelength-conversion loss and degradation of the phosphors shortens the life-time of these LEDs. Moreover, a conventional phosphor converting (PC) white LED is composed of two-color spectra such as blue and yellow, hence, a low color rendering index (CRI) is inevitable. Furthermore, the accumulated heat in the device gradually deteriorates the quality of the phosphor^1^. To overcome these issues, many groups have researched InGaN-based phosphor-free mixed color white-light emitters. As a general approach, phosphor-free white LEDs are fabricated by stacking blue- and yellow emitting active layers on an n-GaN template grown on a sapphire substrate^2^,^3^,^4^. However, the optical power of the yellow-emitting quantum wells (QWs) is low owing to the low quality of the In-rich InGaN layers. To address this concern, a quantum dots (QDs)-in-QW structure has been investigated as an active region^5^,^6^. However, the techniques for growing high-quality QDs embedded in QWs are very complicated. A three-dimensional (3D) multifaceted GaN structure obtained by selective area growth (SAG) effectively achieved highly-efficient long wavelengths and white emissions, which were reported from structures such as prisms, hexagonal-pyramids, annular-circles, and trapezoidal n-GaN arrays^7^,^8^,^9^,^10^. Multi-faceted structures such as polar and semi-polar crystal planes were formed by 3D GaN overgrowth on the mask. When InGaN/GaN QWs were monolithically grown on the multi-faceted GaN structure, different indium compositions and QWs of different thicknesses were formed on each facet, causing the emission of mixed color light. However, this technique needs additional photolithographic processing to make a patterned SiO_2_ mask on a GaN template or substrate before growing the GaN multiple facet structures. Furthermore, it is difficult to make a flat p-type GaN (P-GaN) layer for adequate electrode contact on the top of the LED. In general, a silicon nitride (SiN_x_) interlayer is used as an *in-situ* nanomask in metal organic chemical vapor deposition (MOCVD) to decrease defects in the GaN template^11^,^12^,^13^,^14^. We expected that, if adequate *in-situ* growth conditions were used in MOCVD, this nanomask could lead to 3D GaN nanostructures consisting of nanosized multiple facets. In this study, we introduced an *in-situ* SiN_x_ interlayer to grow GaN nanosized multiple facets formed by 3D GaN overgrown on it. The nano-sized polar and semi-polar planes of the self-organized GaN multiple facets induced different concentrations of indium and various thicknesses of InGaN on each facet, when an InGaN/GaN double-hetero (DH) structure was grown on them. As a result, a monolithic phosphor-free white light emitter was fabricated using MOCVD.

## Results and Discussion

### Surface morphologies of InGaN templates

**Figure 1:**
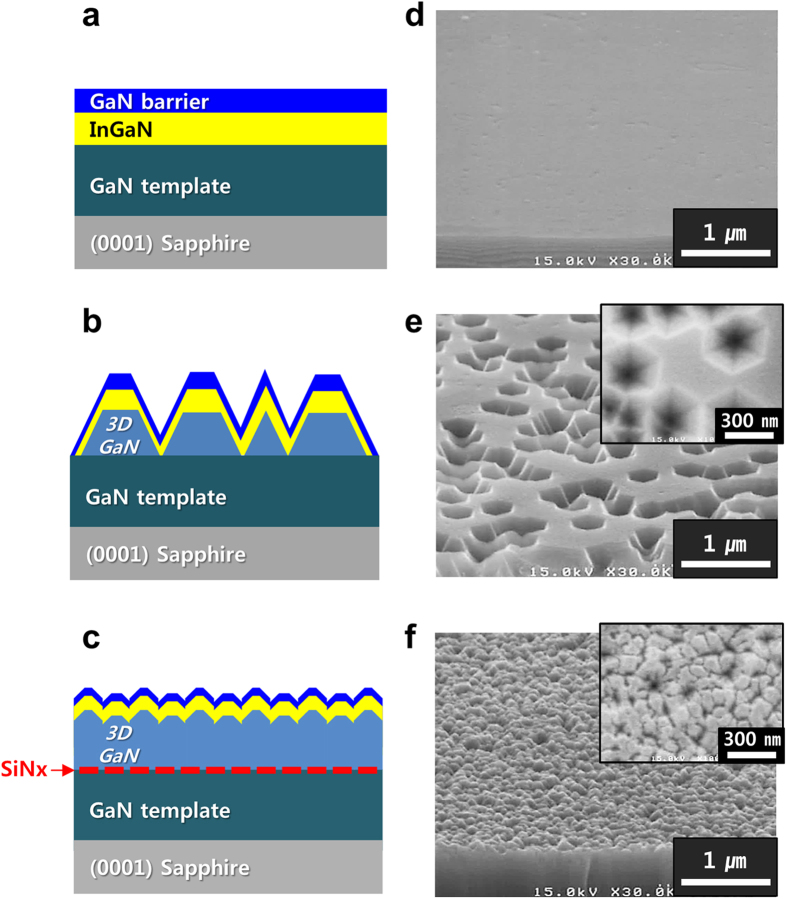
Schematics and SEM images of three samples. (**a,d**) sample-A, (**b**,**e**) sample-B, and (**c**,**f**) sample-C, respectively.

**Figure 2:**
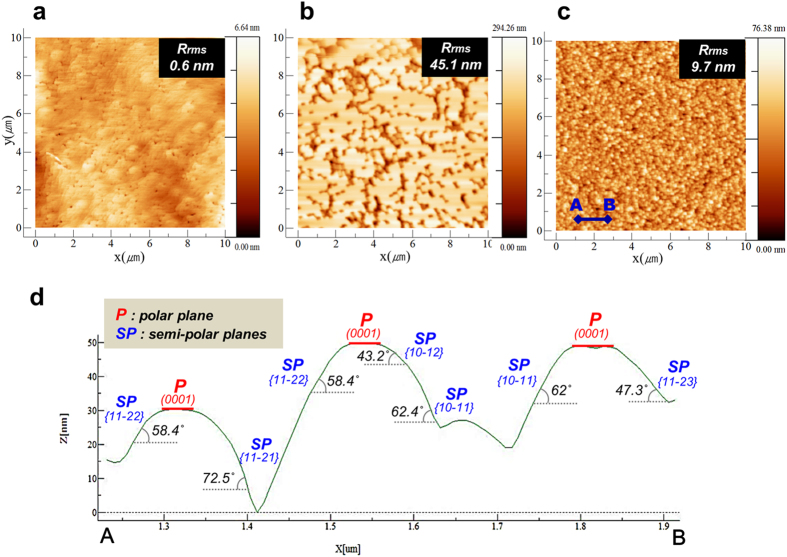
AFM measurement. (**a–c**) Surface images with the values of root-mean-square roughness of sample-A, sample-B, and sample-C, respectively. (scan area: 10 μm × 10 μm). (**d**) AFM line scan profiles between position A and position B in sample-C.

Initially, we fabricated three InGaN/GaN templates on a c-plane sapphire substrate using MOCVD, as shown in Fig. 1(a–c). The three InGaN/GaN templates were named sample-A, sample-B, and sample-C. Sample-A and sample-B were prepared for comparison with sample-C. Figure 1(d–f) show the scanning electron microscopy (SEM) surface morphology of the three InGaN/GaN templates. While sample-A had a flat surface, sample-B showed numerous randomly distributed inverted-pyramid-pits (IPPs). This is because the migration length of Ga adatoms is shorter under the low-temperature growth condition, which results in the growth of 3D GaN layers on the GaN template. The average diameter, height, and density of the IPPs were approximately 300 nm, 260 nm, and 3 × 10^9^/cm^2^, respectively. In addition, sample-B exhibited two crystal planes of the GaN surface: (0001) [flat regions] and {10–11} [inclined regions of IPPs]. In general, the vertices of IPPs are directly connected to the threading dislocations (TDs) propagating from the interface between the GaN layer and the sapphire substrate^15^,^16^. Sample-C, on the other hand showed high-density GaN nanostructures on the surface, whose diameter, height, and density were approximately 95 nm, 30 nm, and 1 × 10^10^/cm^2^, respectively. Moreover, the GaN nanostructures on sample-C fairly uniformly distributed in comparison with sample-B, which we expected to be formed by the SAG of 3D GaN on the SiN_x_ nanomask^17^. Figure 2(a–c) show the atomic force microscopy (AFM) results for 10 μm × 10 μm area. It is clear from Fig. 2(a), that sample-A showed the lowest value of root-mean-square (RMS) roughness (0.6 nm) among the three samples, which indicates that the InGaN/GaN DH layer was grown on the GaN template in a well-ordered fashion without any degradation of the InGaN surface. In contrast, sample-B shows the highest value of RMS roughness (45.1 nm), owing to high surface fluctuations caused by multiple IPPs on the surface. Sample-C exhibits a lower RMS roughness (9.7 nm) than that of sample-B and a uniform distribution of GaN nanostructures, as shown in Fig. 2(c). Figure 2(d) shows a cross-sectional line-scan profile of sample-C along the blue solid line, marked A-B in the lower left corner of Fig. 2(c); the scan distance was approximately 0.7 μm. The profile result shows diverse polar and semi-polar multiple-facets of GaN formed by 3D GaN overgrowth on the nano-sized window and mask area of the SiN_x_ interlayer. In particular, it shows the semi-polar facets consisting of several crystal planes such as {10–11}, {11–22}, {11–21} and {10–12} which can be deduced using the angle difference between (0001) c-plane and each semi-polar plane; the InGaN/GaN DH structure was also grown on these multiple facets.

### InGaN DH structure on nano-sized multiple facets

**Figure 3:**
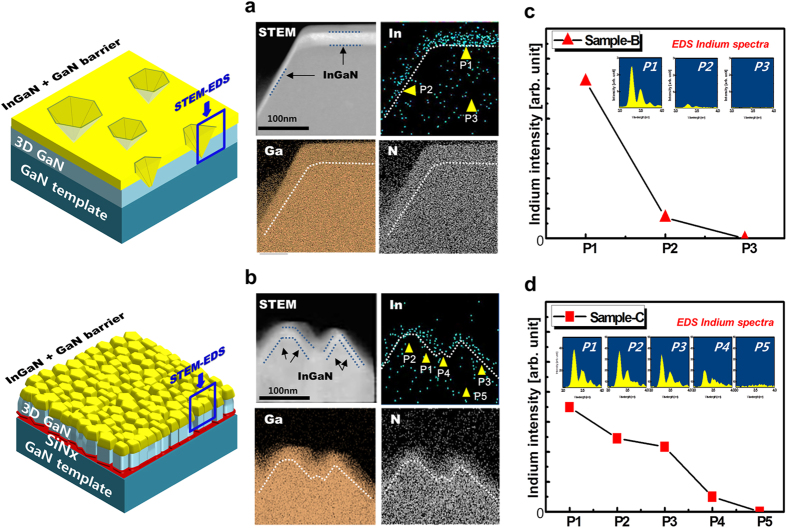
Cross-sectional STEM-EDS characterization. (**a,b**) Cross-sectional STEM-EDS mapping images of sample-B and sample-C, respectively. (**c,d**) EDS spot spectra showing change of indium content in sample-B and sample-C, respectively.

Figure 3(a,b) show the cross-sectional scanning transmission electron microscopy (STEM) and energy-dispersive x-ray spectroscopy (EDS) mapping images of the atomic distributions of gallium, indium, and nitrogen in the indicated regions of sample-B and sample-C, respectively. Figure 3(a) clearly shows that the InGaN layer was grown on the flat plane (0001) and inclined plane {101–1} of the IPPs in sample-B. The white dashed line indicates the interface boundary between the re-grown 3D GaN and the InGaN layer. According to the STEM image, the InGaN layer on the (0001) plane is thicker than that on {10–11}, owing to the relatively shorter diffusivity of indium adatoms on the (0001) plane compared to {10–11}. Moreover, the diffusivity of indium, due to high vapor pressure, is higher than that of gallium^18^. Therefore, the indium distribution is more dependent on multiple facets of underlying GaN than that of gallium. The EDS spot analysis was carried out to investigate the indium concentration of InGaN on each facet. The points are denoted as P1, P2, and P3 in the indium EDS mapping image using short yellow arrows. The point spectra indicate that the indium content at P1, P2, and P3 is approximately 10.5%, 1.4% and 0%, respectively, as shown in Fig. 3(c). As a result, in the case of sample-B, the thick InGaN layer grown on (0001) exhibited higher indium concentration than that on {10–11}. Figure 3(d) shows the cross-sectional STEM-EDS mapping images of sample-C. As shown in the indium STEM image, the InGaN layer is also distributed along the nanosized multiple-facets of GaN whose positions are indicated by yellow arrows and blue dashed lines. Sample C exhibited more GaN crystal planes than sample B. To investigate the indium concentration of InGaN on each facet, the point spot spectra were also measured. The result indicate that the indium concentrations at P1, P2, P3, P4 and P5 were approximately 3.5%, 2.5%, 2.2%, 0.5%, and 0%, respectively. It should be noted that the indium distribution is not quantitative but can be relatively compared in the InGaN layer. Sample-C showed more variation in indium distribution along the underlying GaN multiple facets than sample-B; this can be attributed to more diverse multiple facets than sample-B^19^. The highest indium concentration in the InGaN layer of sample-C occured at P1 (semi-polar facet) in Fig. 3(d), which also showed the highest value of InGaN thickness as shown in Fig. 3(b). In addition, the InGaN thickness also varied with the nanosized multiple-facets of GaN as shown in Fig. 3(b,d). To summarize: there was more variation of indium concentration along the high density and nano-sized GaN multi-facets of sample-C than on the GaN micro-facets of sample-B.

**Figure 4:**
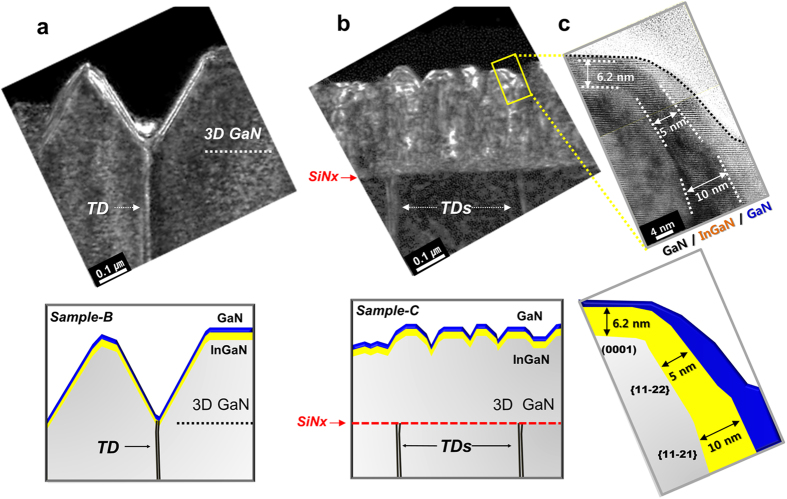
Cross-sectional TEM analyses. (**a**,**b**) Cross-sectional TEM images and each illustration of sample-B and sample-C. The electron diffraction is along z = [1–100] and g = [11–20]. (**c**) HR-TEM image and illustration of yellow rectangular region in sample-C. The result indicated InGaN with various thicknesses was grown on multiple facets of GaN nanostructures. (Yellow color region in illustration).

Figure 4(a,b) shows the cross-sectional TEM images of sample-B and sample-C, respectively. The electron diffraction is along z = [1–100] with g = [11–20], and it should be noted that only edge-type and mixed-type dislocations are observable in this condition. The white dashed line in Fig. 4(a) indicates the boundary between the GaN template and the regrown 3D GaN layer. As shown in Fig. 4(a), the TDs generated at the interface between the GaN template and the sapphire substrate was directly connected to with the vertex of the IPPs and to the InGaN/GaN DH active layer. On the other hand, Fig. 4(b) shows that the TDs were blocked by the SiN_x_ interlayer^11^,^12^,^13^,^14^. The uppermost profile of sample-C demonstrates undulated GaN multi-facets in accordance with the AFM line-scan profile in Fig. 2(d). The shape of the re-grown 3D GaN on the SiN_x_ interlayers appears as connected GaN-nano-columns (GNCs). We speculate that the GNCs were primarily grown along the vertical direction at the openings of the SiN_x_ interlayer. Although some dark regions are exhibited in Fig. 4(b), obvious vertical grain boundaries between the GNCs cannot be seen. It is believed that the GNCs were partially merged with each other at the m-planes of GaN during the 3D GaN re-growth. Figure 4(c) shows the HR-TEM image of the yellow rectangular area in Fig. 4(b); dark regions (InGaN) and bright regions (GaN) are evident, and the position of the dark regions corresponds to that of the InGaN layer in the STEM-EDS mapping images, as shown in Fig. 3(b)^6^,^20^,^21^,^22^. Figure 4(c) also shows that the InGaN/GaN DH layer grew on multiple GaN facets, including (0001), {11–22}, and {11–21}, with thickness of approximately, 6.2 nm, 5 nm, and 10 nm, respectively. As mentioned above, the thickness variation of InGaN depends on the diffusivity of indium and gallium adatoms at each GaN facet^9^,^18^,^23^; the results shown in Fig. 4(c) confirm this.

### Photoluminescence analysis of InGaN templates

**Figure 5:**
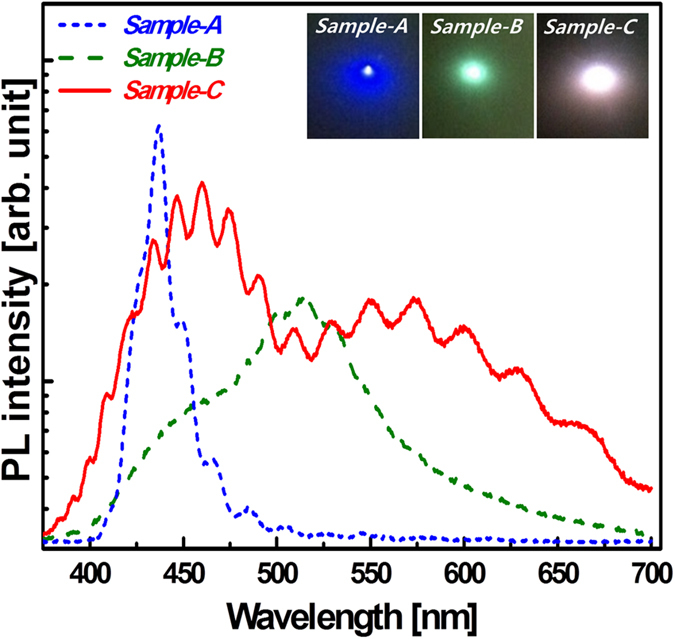
Room temperature PL spectra. Insets exhibit light emitting image of each sample.

Figure 5 shows the room temperature photoluminescence (PL) spectra of three samples. The InGaN/GaN DH structures were grown at the same time on three GaN templates at 750 °С, and the inset shows PL emission images of each sample. Sample-A, indicated by blue dashed line, showed blue emission with a single peak spectrum centered at of 437 nm. Sample-B, indicated by the green dashed line, showed broad green emission with peaks at 456 nm and 514 nm. We believe that the short and long wavelengths in sample-B were generated from InGaN DH structures grown on the {10–11} and the (0001) planes, respectively, which are in good agreement with the STEM mapping results in Fig. 3(a). The peak-intensity was lower than that of sample-A because of the high non-recombination rate at the IPPs. Sample-C, indicated by a red solid line in Fig. 5, showed clearly separated, relatively broad, peaks at 460 nm and 574 nm. We believe that this broadness was caused by the variations in indium concentration and InGaN thickness on GaN facets, in accordance with the STEM and HR-TEM. From these results, we speculate that the blue and yellow emissions mainly arose from InGaN DH structures grown on {11–22} and {10–11} facets, and on (0001) and {11–21} facets, respectively, probably due to variation of InGaN thickness on each facet. In addition, it should be noted that the emission intensity of sample-C was higher than that of sample-B; this can be attributed to the increased light extraction efficiency (LEE) from the high-density nanostructure formed on sample-C, and also to the reduction in defects that results from the use of the SiN_x_ interlayer.

### Surface morphology and electroluminescence of the phosphor-free white LED

**Figure 6:**
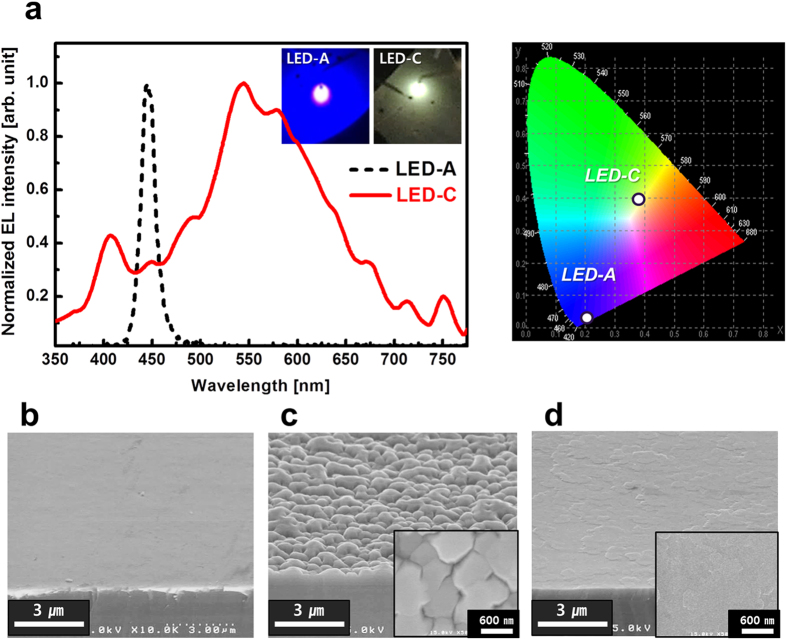
EL spectra and SEM surface morphologies of LEDs. (**a**) EL spectra of LED-A and LED-C, respectively. (No luminescence was observed from LED-B). Insets exhibit EL emission images of LED-A and LED-C, respectively. The color coordinates of EL spectra of LED-A and LED-C were indicated at the Commission Internationale de I’Eclairage (CIE) 1931 chromaticity diagram. (**b–d**) The SEM images showing top surface morphologies of LED-A, LED-B, and LED-C, respectively. Insets show the magnified plane-view SEM images, respectively.

Figure 6(a) shows the electroluminescence (EL) results of three LEDs, and Fig. 6(b–d) show the corresponding SEM surface images of top-most P-GaN layer. All three InGaN DH LEDs were grown under identical conditions. As shown in Fig. 6(a), LED-A and LED-C showed blue and white emissions, respectively, at a current of 20 mA, whereas no emission was observed from LED-B. It is obvious that a huge current leakage occurred through the unmerged IPPs of LED-B, [shown in the magnified inset image of Fig. 6(c)] because the TDs directly connected to pits act as current leakage paths in thin films^16^,^17^. In contrast, LED-C showed a relatively flat P-GaN surface in comparison with that of LED-B, because its nanosized multiple facets were fully merged within the 0.12-μm-thick P-GaN layer. The slightly rough surface shape exhibited by LED-C was due to the different heights of the GaN nanostructures. This surface could be improved even further by P-GaN growth optimization. While LED-A showed blue emission with λ_p_ = 445 nm, sample-C showed a broad spectrum with a range of 400–700 nm, as illustrated by the warm white emission image in the inset of Fig. 6(a). Our results demonstrate that *in-situ* InGaN/GaN nanostructures formed on a SiN_x_ interlayer are an effective way to achieve a broad wavelength range of light emission using only MOCVD growth technology without any external processes.

## Conclusions

We note that there are many factors that could improve the optical performance of PF-white LEDs, including the SiN_x_ interlayer density, the dimensions of the GaN nanostructures, the structure of the active region, and the growth conditions of P-type GaN. Our future work will focus on growth optimization. In this study, we demonstrated a novel and practical method for growing phosphor-free white-light emitters without any external processing, using only *in-situ* high-density GaN nanostructures formed by MOCVD growth over a SiN_x_ interlayer. The polar and semi-polar facets formed on GaN nanostructures induced various indium concentrations and InGaN thicknesses on the facets when InGaN/GaN DH was grown on them. As a result, a monolithic white-light emitter was achieved by a single-step growing process using MOCVD. We suggest that this technique can be applied to a large-diameter wafer to fabricate phosphor-free white light emitters.

## Methods

### Growth of InGaN templates and LEDs

We used a Thomas-Swan close-coupled showerhead (CCS-MOCVD) reactor system to grow InGaN templates and LEDs. After annealing the sapphire substrate in H_2_ at 1080 °С for 5 min, a 30-nm-thick GaN buffer layer was deposited by MOCVD at 500 °С, followed by deposition of a 2-μm-thick undoped GaN(u-GaN) layer at 1060 °С. After bringing the temperature down to 860 °С, a SiN_x_ layer was deposited on the u-GaN template in H_2_ for 1500 s, followed by the growth of a 0.4-μm-thick 3D u-GaN structure at 750 °С. An InGaN/GaN DH structure was grown on the template with a thickness of 20 nm and a GaN thickness of 9 nm. Finally, three LEDs (LED-A, LED-B, and LED-C), each containing a 3-μm-thick n-type GaN template and a 0.12-μm-thick p-type GaN cladding layer were grown by MOCVD using, trimethylindium (TMIn), trimethylgallium (TMGa), silane (SiH_4_) and ammonia (NH_3_) as precursors for indium, gallium, silicon and nitrogen, respectively. Silane (SiH_4_) and bis(cyclopentadienyl) magnesium (Cp_2_Mg) were used as n-type and p-type dopant sources, respectively.

### Characterizations

The surface morphology of the samples was measured by SEM (Hitach-4000) and AFM (My-scope plus, Nanofocus Co.). The cross-sectional microstructure and distribution of the indium concentration of the samples were analyzed by TEM (JEM-2100 F, JEOL, 300 KeV) and EDS with STEM (JEM-2100 F, JEOL, 200 KeV and Cs corrector, CEOS), respectively. The TEM specimens were prepared by using a focused ion beam system (NOVA 600 Nanolab). Also, PL (He-Cd laser 325 nm) and EL (ELT-1100 LED Chip tester) were used to determine the optical properties at 300 K of the InGaN templates and the LEDs, respectively.

## Additional Information

**How to cite this article**: Min, D. *et al.* Phosphor-free white-light emitters using *in-situ* GaN nanostructures grown by metal organic chemical vapor deposition. *Sci. Rep.* **5**, 17372; doi: 10.1038/srep17372 (2015).
